# Study on the Influence of Sn58Bi Alloy on Rock Perforation Plugging Performance

**DOI:** 10.3390/ma18061195

**Published:** 2025-03-07

**Authors:** Chunqing Zha, Wenhe Xia, Wei Wang, Gonghui Liu, Jun Li, Wei Liu

**Affiliations:** 1 College of Mechanical and Energy Engineering, Beijing University of Technology, Beijing 100124, China; chachunqing@bjut.edu.cn (C.Z.); xiawenhe@emails.bjut.edu.cn (W.X.); lgh_1029@163.com (G.L.); 2 School of Engineering, China University of Petroleum (Beijing) at Karamay, Karamay 834000, China; lijun446@vip.163.com; 3 CNPC Engineering Technology R&D Company Limited, Beijing 102206, China; liuweidri@cnpc.com.cn

**Keywords:** Sn58Bi alloy, rock perforation plugging, mechanical plug, liquid plug

## Abstract

In order to solve certain issues, such as brittle fracture corrosion and easy failure, which occur under high ambient temperatures and high breakthrough pressures when conventional cement is used to plug rock perforations, a method using a Sn58Bi alloy was adopted in this paper; it was utilized to melt and plug a perforation. Subsequently, the influence of the characteristics of the rock perforation (such as perforation length and diameter) on alloy plugging performance under different conditions and ambient temperatures was studied. The experimental results show that the plugging effect of the Sn58Bi alloy was affected by ambient temperature, plugging diameter, and length. When the plugging length was 100 mm and the perforation diameter was 10 mm, the mechanical plug performance decreased by 24.0% when the ambient temperature increased from 30 °C to 60 °C, and then decreased by 19.0% when the ambient temperature increased to 90 °C. At 30 °C, the mechanical plug performance decreased by 30.4% when the diameter decreased from 10 mm to 8 mm, and decreased by 28.0% when the diameter decreased to 6 mm. When the length was constant and the diameter was decreased from 10 mm to 8 mm and then to 6 mm, the hydraulic plugging effect became better, and the trend increased from 33.7% to 37.2%.

## 1. Introduction

In oil and gas well exploitation, perforation is an important part of the oil exploitation stage. It produces micro-cracks or -pores in the rock, increases permeability, and makes it easier for oil and gas to flow into the oil well to help in the exploration and exploitation of oil [1]. In order to improve the efficiency and quality of oil production, it is necessary to block perforation in low-yield or structurally unstable reservoirs; in doing so, one can prevent reservoir pressure loss caused by water channeling and gas channeling, so as to ensure oil and gas recovery and production continuity. At the same time, it also prevents oil and natural gas from leaking into the surrounding strata through perforation or casings, affecting the surrounding formation and biological environment [2,3]. Therefore, the study of oil well plugging, especially rock perforation plugging, is particularly important. The key to oil well plugging is to use a plugging material to plug the perforation. Its plugging effect is related to the quality, integrity, and environmental safety of the underground operation. Therefore, it is of high research significance to realize high-quality perforation plugging.

At present, cement is the most commonly used underground plugging material. Cement has the advantages of low cost and simple operation. However, cement has the disadvantages of poor fluidity, brittle fracture, and poor corrosion resistance. It may fail under high ambient temperatures and high stress, and there may be obvious shrinkage and poor interface bonding over time. At the same time, the change in the underground environment can cause cracks or cavities/micro-annuli to occur inside the cement. Therefore, the use of cement cannot guarantee the long-term integrity of oil well plugs in well construction and abandonment operations [4,5,6]. Therefore, it is important to find high-strength materials that have good fluidity and corrosion resistance. New plugging materials also need to have a minimal impact on the environment, which can be achieved through different plugging scenarios while simultaneously enhancing oil well stability.

The bismuth–tin (BiSn) metal alloy has been gradually proven to be a substitute for cement plugs over the past few years because of its advantages: safety, environmental protection, and low cost. The most important reason for the use of a BiSn alloy is that it has a lower melting point (138 °C) relative to other bismuth metals (such as BiAg’s melting point, 180 °C, and BiCu’s melting point, 271 °C), and exhibits micro-expansion and compactness during solidification. In addition, it is a eutectic metal that bypasses the gel phase during solidification and directly changes from the liquid phase to the solid phase. The BiSn alloy takes advantage of the expansion characteristics of bismuth during solidification, which results in a good plugging effect between the alloy and the casing.

In order to explore the actual influence of BiSn alloy material on plugging, Jia Zili and Shi Bin used numerical simulations to calculate different well tracks and optimize them to reduce oil and gas flow losses [7]. Paul John Carragher et al. proposed a method which used low-melting-point alloys to achieve oil well plugging [8]. Through experimental comparison, Manataki and Zhang proved that bismuth plugs have a higher strength than cement plugs in a combination experiment with steel and formation, and that bismuth plugs have a better plugging effect [9,10]. Lewaa Hmadeh et al. verified through experiments that alloy plugs have good mechanical pressure bearing performance and can withstand large axial loads [11]. Lewaa Hmadeh and Marcelo Anunciação Jaculli et al. also studied the effect of ambient temperature on the performance of plugs made of eutectic BiSn alloy [12]. The above research proves that the BiSn alloy material exhibits good pressure resistance, stability, and plugging effects in perforation plugging, but further research is needed on the influence of its performance and parameters in rock perforation. This is because, when plugging a rock perforation, the micro-expansions generated during the solidification of a BiSn alloy may cause friction between the perforated rock wall surfaces and may impact the plugging effect. The issues that remain to be solved in practical applications include the integrity of alloy plugging, impermeability, and mechanical load resistance, as well as understanding parameter changes, such as ambient temperature and pressure. Additionally, ambient temperature and pore sizes in rock perforation also affect the plugging effect. Therefore, the main purpose of this study is to research the influence of perforation size parameters and ambient temperature on the perforation performance of an BiSn alloy in fused and sealed rock.

In order to analyze the plugging load and the influencing factors of the material during perforation plugging, an experimental method of injecting a liquid BiSn alloy into rock to simulate the plugging of perforations is proposed in this paper. A series of detection devices for mechanical pressure bearing capacity and liquid plugging in rock perforations is developed. The changes in plugging performance between the alloy and the rock under different ambient temperatures and perforation diameters and lengths were analyzed. At the same time, the influence of the structure on plugging performance was analyzed using an optical microscope. The influence of alloy plugging in different rock perforations and at different ambient temperatures is also studied. The research in this paper can provide a new theory and solution for BiSn alloy plugging for rock perforations.

## 2. Alloy Properties and the Principle of Perforation Plugging in Rock

### 2.1. Alloy Characteristics

In this study, the alloy used was Sn58Bi, a low-melting-point alloy, which was an eutectic alloy composed of 58% bismuth and 42% tin. The basic characteristics of the alloy are shown in Table 1.

The plugging material has the following advantages [15,16]:

(1) Low melting point: The energy demand for the melting process is relatively low.
(2) Fluidity: It is capable of flowing into the minute gaps and irregular geometries within the perforation.
(3) Expansion: Upon solidification from the liquid state to the solid state, the alloy undergoes volume expansion, which is beneficial for perforation plugging.
(4) High density: Owing to its high density, when the alloy flows into the perforation, low-density substances such as impurities or gases can be buoyed to the upper end. They become attached to the upper surface of the rock and are displaced. This phenomenon facilitates the formation of a pure alloy plug and enables the achievement of plug integrity.
(5) Corrosion resistance: The alloy exhibits resistance to corrosion, particularly in the presence of gases such as H_2_S and CO_2_.

### 2.2. Alloy Plugging Method of Perforation

**Figure 1:**
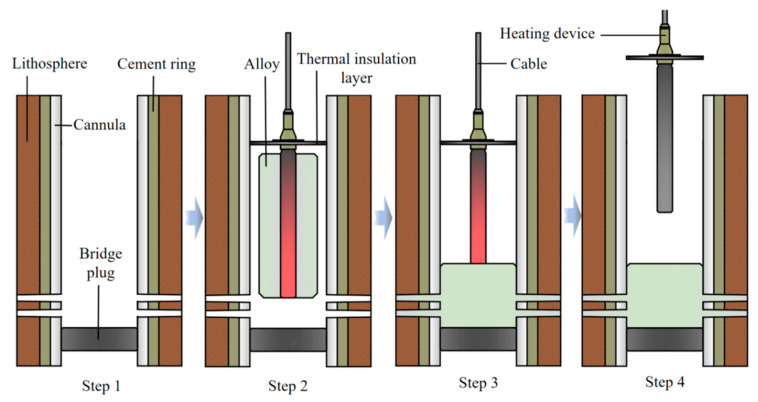
A schematic diagram of heating device for molten alloy plugging perforation.

**Figure 2:**
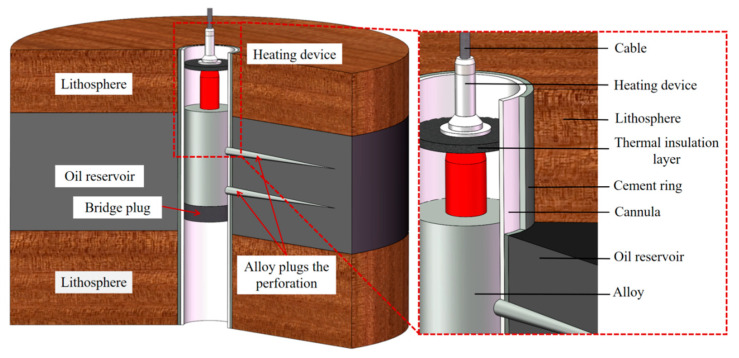
A schematic diagram of alloy plugging perforation.

When plugging the perforation, the heating device surrounding the low-melting-point Sn58Bi alloy was positioned at the specified location (as depicted in Figure 1 and Figure 2). After positioning the bridge plug, the heating device situated above it was switched on to melt the alloy. Subsequently, an overlying pressure was applied to the molten alloy, ensuring that the alloy fully flowed into the perforation. Before the alloy solidified, the device was removed, as shown in Figure 1. This method offers the advantages of simple operation, high plugging efficiency, and low environmental pollution.

**Figure 3:**
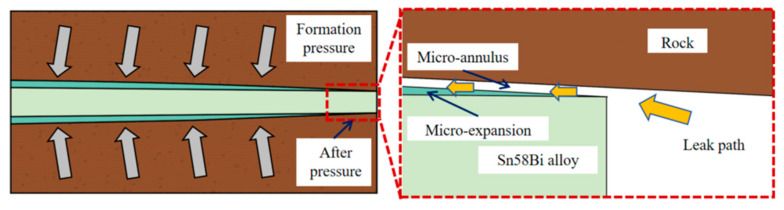
A schematic diagram of alloy pressure plugging perforation.

Figure 3 illustrates the detailed state of the close combination between the Sn58Bi alloy and the rock perforation, subsequent to the micro-expansion phenomenon during alloy solidification. Nevertheless, the rock layer or formation liquid continuously exerted high pressure on the alloy plug used for plugging. As a result, the alloy plug underwent deformation and displacement, leading to the failure of the plugging and ultimately resulting in the leakage of the oil well liquid or gas. Consequently, to measure and analyze the limit value of the plugging performance of the Sn58Bi alloy, a mechanical plug-testing device and a hydraulic plug-testing device were developed. These devices are capable of studying the mechanical and liquid-tight plugging performance of the perforation under diverse conditions. Additionally, the micro-structure was analyzed. 

## 3. Experimental Device

### 3.1. Preparation of Alloy in Rock Perforation

**Figure 4:**
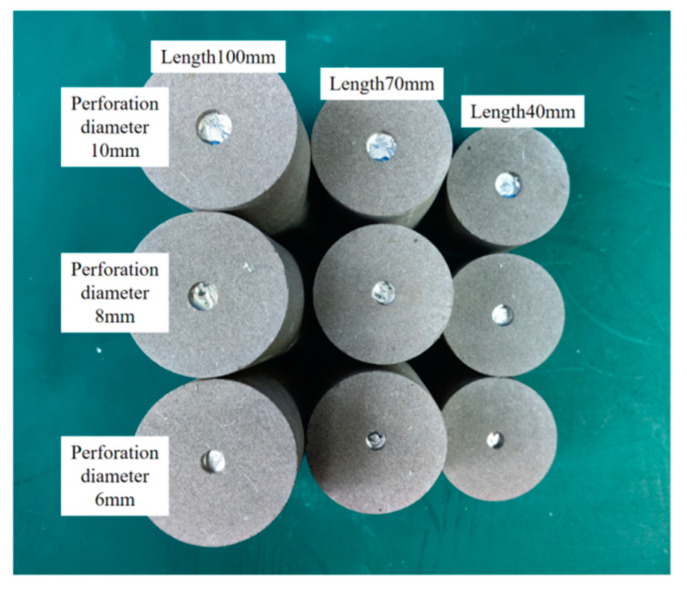
Rocks of different sizes used in the experiment.

The rock was sandstone with a diameter of 50 mm, and lengths of 100 mm, 80 mm, and 60 mm, and perforation diameters of 10 mm, 8 mm, and 6 mm were used. The alloy was injected into the rock (as shown in Figure 4).

### 3.2. A Device for Mechanical Push-Out Experiment

**Figure 5:**
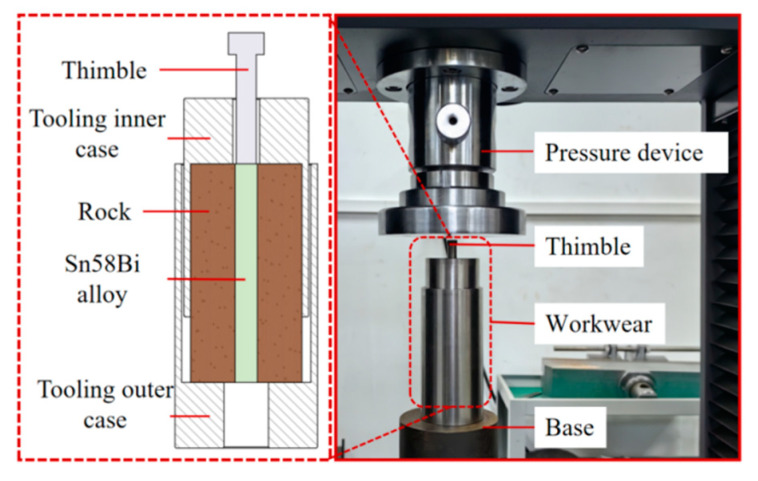
The schematic and physical diagram mechanical push-out experiment.

To investigate the mechanical plugging capacity of the alloy utilized for perforation plugging under the impact of the external environment, a set of mechanical push-out experimental apparatus was devised. The schematic illustration of this experimental apparatus is depicted in Figure 5. The experimental setup encompasses a bespoke experimental unit and a universal testing machine. The bespoke experimental unit comprises tooling, sandstone samples, and the alloy. The tooling is partitioned into an inner shell of the tooling and an outer shell of the tooling.

### 3.3. A Device for the Hydraulic Plugging Pressure Experiment

To study the hydraulic plugging ability of the alloy for perforation plugging under the influence of the external environment, a set of hydraulic sealing experimental devices was designed. The hydraulic plugging experimental setup consists of an experimental module, a temperature-control box, and a hydraulic pump. The experimental module is composed of a plugging end-cover, a high-ambient-temperature-resistant plugging ring, a rock casing, sandstone specimens, and the alloy. During the hydraulic plugging experiment, the bottom of the device is connected to the outlet of the electro-hydraulic press via a hydraulic valve. After the hydraulic press is filled with water, the pressure-relief port of the pressure-relief valve is connected to a water tank.

**Figure 6:**
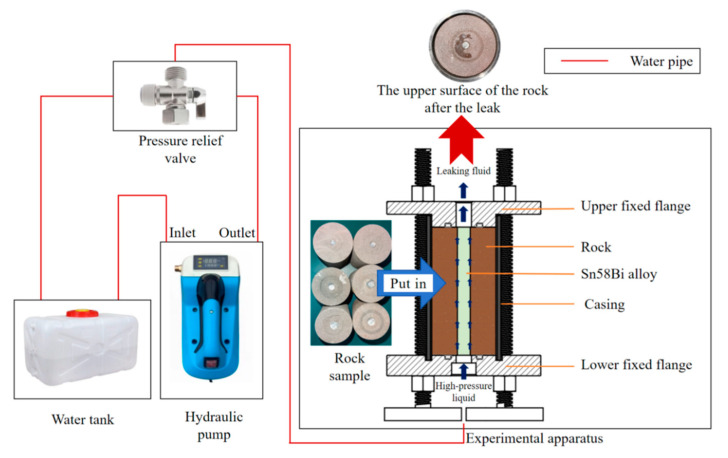
Connection diagram of hydraulic plugging experimental device.

**Figure 7:**
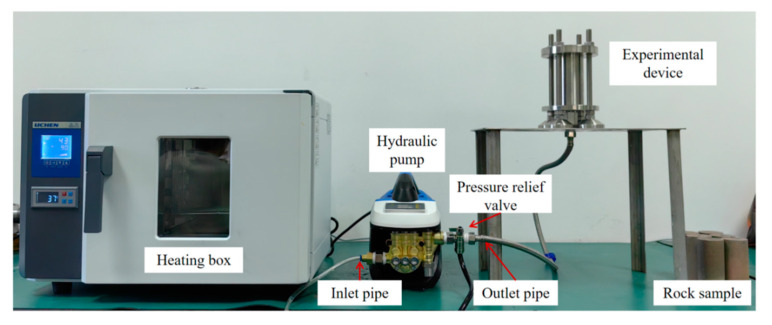
The physical picture of the device in the experiment.

The hydraulic plugging experimental apparatus was configured with an upper fixed flange, a casing, a rock specimen, a lower fixed flange, and supporting screws. The casing was joined to the groove of the lower fixed flange. Subsequently, the rock specimen was positioned on the lower fixed flange through the casing. The pressure-applying device was connected to the upper fixed flange via a high-pressure pipe, while the liquid detection device was attached to the lower fixed flange, as depicted in Figure 6 and Figure 7.

## 4. Study on the Properties of Alloy Plugging Rock Perforation

### 4.1. Mechanical Push-Out Experiment

**Figure 8:**
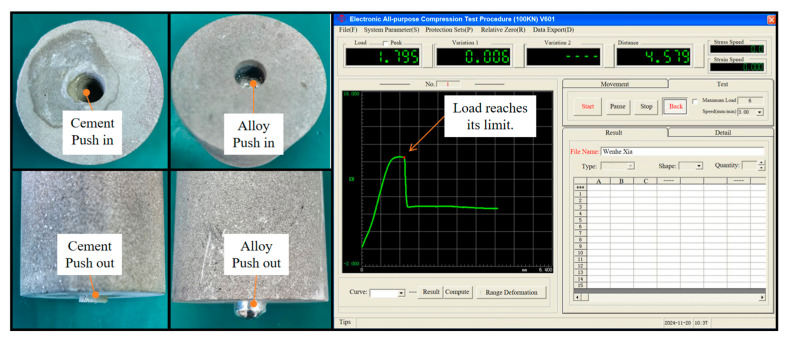
Cement and alloy plugging rock before and after the experiment and the experimental operation interface.

To investigate the plugging effect of the alloy in rock perforations, a mechanical push-out experiment was conducted, as illustrated in Figure 8. The experimental procedure is as follows: First, the rock perforations were filled with either cement or the alloy separately. After the filling materials had solidified and formed, the specimens were placed into the tooling for the mechanical push-out experiment. During the experiment, the push-out pressure was gradually increased (with the advancing speed of the thimble set at 3 mm/min), and the corresponding values were observed. As the pressure was increased, the pressure–displacement curve typically showed a gradually upward trend. However, when a sharp change in pressure occurred, the pressure application was halted. Subsequently, data were recorded, and relevant images were generated. Finally, to explore the influence of different factors, the size of the rock perforations and the ambient temperature were varied, and the above-mentioned experimental operations were repeated.

As depicted in Figure 8, once the load force exceeded the frictional force between the rock and the alloy, the alloy began to move, thereby rupturing the mechanical plug formed between the rock and the alloy.

### 4.2. Hydraulic Plugging Pressure Experiment

To mimic the actual perforation environment, high-pressure liquid was directed into the container from one side of the plugging region. Meanwhile, the other side of the device was linked to a hydraulic pressure device. In the event that the liquid penetrated the interface between the alloy and the rock perforation, it would overflow through the outlet located above the device. Once this occurred, the experiment would be terminated, and the current pressure value would be regarded as the maximum breakthrough pressure.

Prior to conducting the plugging experiment, the liquid-tightness of the experimental device was examined. Initially, the liquid was injected into the rock perforations that had been plugged with the alloy. Subsequently, the pressurization process was gradually initiated. As water seeped through the gap between the rock perforations and the alloy, it indicated that a leakage had occurred. Consequently, the experiment for this particular group was terminated.

**Figure 9:**
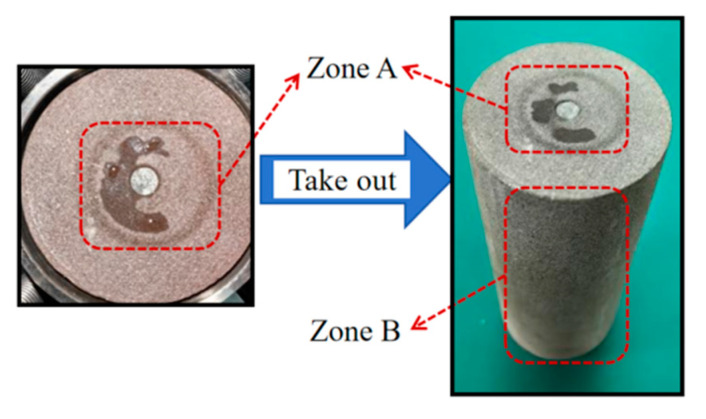
The actual rock surface condition.

After disassembling the device, the liquid leakage on the rock surface was observed, as presented in Figure 9. On the upper surface of the rock, outside the plugging ring (Zone A), no water stains were detected. Similarly, around the rock (Zone B), no water stains were present. This observation indicated that the path of water stains on the upper surface was the micro-annulus between the rock and the alloy. The pressurized liquid had flowed out through the gap between the rock and the alloy. Thus, the liquid-tightness of the experimental device met the experimental requirements.

Subsequently, the formal alloy hydraulic plugging experiment was conducted. The experimental procedures were as follows: The preprepared rock specimens were placed into the experimental apparatus, and the rock liquid-plugging experiment was initiated. During the experiment, the hydraulic pressure was gradually increased, and the corresponding values were carefully observed. Once liquid leakage was detected in the perforation at the bottom of the device, the application of pressure was immediately halted. Then, the data were recorded, and relevant images were generated. To explore the influence of different factors, the size of the rock perforations and the ambient temperature were varied, and the above-mentioned experimental operations were repeated.

## 5. Experimental Analysis

### 5.1. The Influence Factors of Mechanical Pressure of Alloy

#### 5.1.1. Optical Microscope Observation of Alloy and Rock Interface

**Figure 10:**
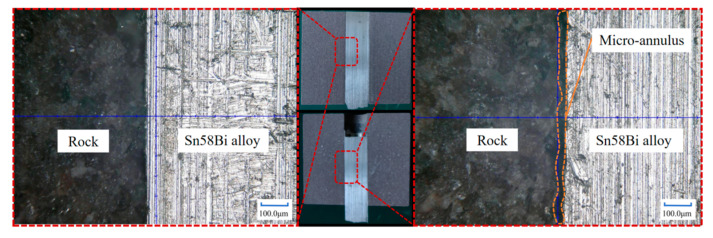
Comparison of the alloy not pushed out and pushed out, as observed by optical microscope.

Upon completion of the push-out experiment, the sample blocks were longitudinally sectioned and subsequently observed under a microscope. As illustrated in Figure 10, it was discovered that, in the unpushed sample, there was no discernible gap between the rock and the alloy. In contrast, in the pushed sample, a certain micro-annulus was present between the rock and the alloy. The underlying cause is that, when the alloy was subjected to external force and displaced, the bonding interface between the rock and the alloy was damaged, thereby giving rise to a gap.

The following Table 2 presents the mechanical push-out experimental data of alloy plugging in rock.

#### 5.1.2. Effect of the Diameter of Plugging Rock Perforation on the Mechanical Bearing Capacity of Alloy

**Figure 11:**
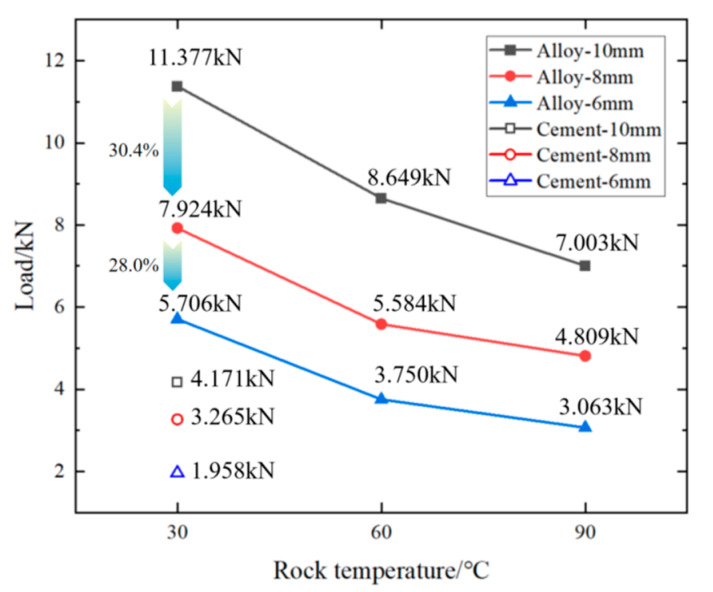
Effect of Sn58Bi alloy and cement on mechanical pressure of plugging rock perforation.

The following figure (Figure 11) shows the relationship between different ambient temperatures (30 °C, 60 °C, 90 °C) and alloy plugging diameters (10 mm, 8 mm, 6 mm) and the maximum load (kN) at the same rock perforation length (100 mm).

From Figure 11, it can be inferred that, at the same rock perforation length, the mechanical plugging effect of cement is inferior to that of the alloy. As the ambient temperature rises, the compressive load of the alloy plugging diminishes. When the perforation alloy plugging length is 100 mm, the mechanical plugging performance varies from 30.4% to 28.0% as the plugging diameter changes from 10 mm to 8 mm and then to 6 mm. When the rock’s ambient temperature changes from 30 °C to 60 °C and the perforation diameter is 10 mm, the breakthrough pressure that the alloy can withstand in the perforation decreases from 11.377 kN to 8.649 kN, representing a 24.0% reduction. When the rock’s ambient temperature ranges from 60 °C to 90 °C, the breakthrough pressure that the alloy plug can endure in the perforation drops from 8.649 kN to 7.003 kN, with a 19.0% decrease in the breakthrough pressure of the alloy plug in the perforation. When the perforation diameter is 8 mm, the reduction in the breakthrough pressure with the increase in the rock’s ambient temperature from 30 °C to 60 °C is 29.5%, and from 60 °C to 90 °C, it is 13.8%. When the perforation diameter is 6 mm, the decrease in the breakthrough pressure as the rock’s ambient temperature changes from 30 °C to 60 °C is 34.3%, and from 60 °C to 90 °C it is 18.3%. The results demonstrate that, as the ambient temperature increases (from 30 °C to 60 °C and then to 90 °C), the trend of mechanical pressure change becomes more gradual.

**Figure 12:**
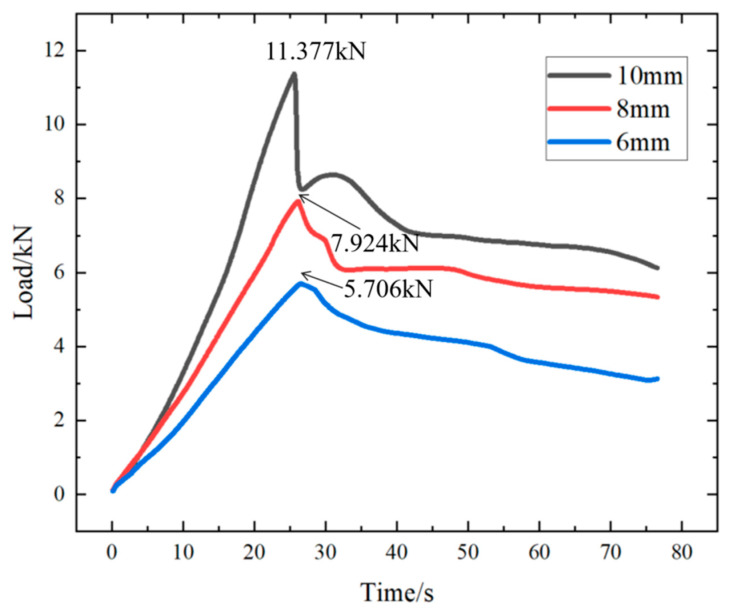
The curve of the load change at 30 °C with time.

In addition, at an ambient temperature of 30 °C and a rock perforation length of 100 mm, the alloy was subjected to external force extrusion, and the curve of the load changing with time was as follows:

As can be observed from Figure 12 above, regardless of the different alloy diameters and diverse rock perforation lengths, the general trend of the load variation with time is approximately consistent. The compressive load of the alloy initially increases and subsequently decreases over time. The peak portion of the curve represents the maximum load at which the alloy plugging fails.

#### 5.1.3. Effect of Ambient Temperature on Mechanical Pressure of Alloy Plug

**Figure 13:**
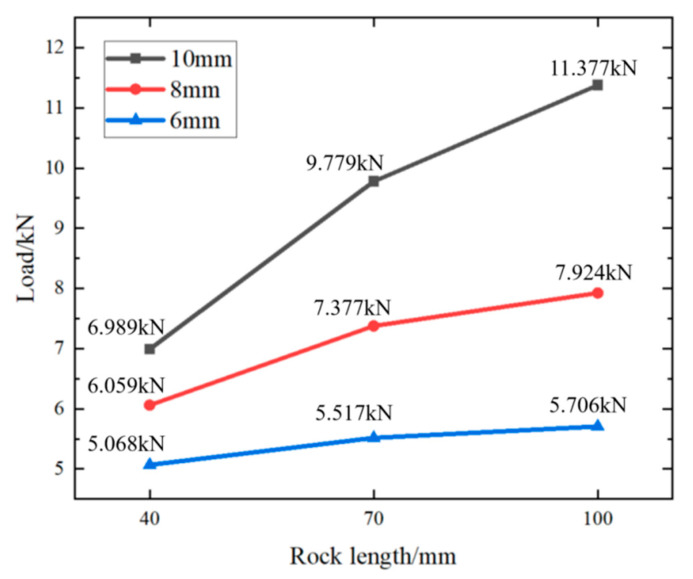
The effect of different factors on the mechanical pressure of Sn58Bi alloy plug at 30 °C.

**Figure 14:**
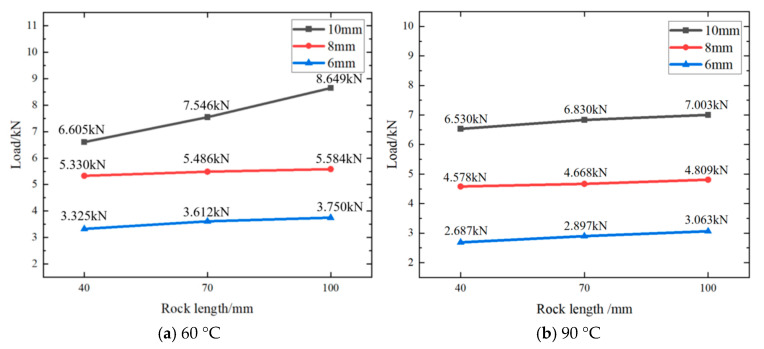
The influence of different factors on the mechanical pressure of Sn58Bi alloy plug at 60 °C and 90 °C.

The following figure (Figure 13) shows the relationship between rock perforation lengths (100 mm, 70 mm, 40 mm), alloy plugging diameters (10 mm, 8 mm, 6 mm), and load (kN) at different ambient temperatures (30 °C (Figure 13), 60 °C (Figure 14a), and 90 °C (Figure 14b)).

As is evident from Figure 13, at a constant ambient temperature, as the plugging length of the rock perforation increases, the plugging performance of the alloy plug improves. Nevertheless, as the length of the alloy-plugged perforation continues to grow, this improvement trend becomes more gradual.

Taking Figure 13 as an illustration, when the ambient temperature of the rock with the alloy was 30 °C and the perforation diameter of the rock was 10 mm, as the plugging length increased from 40 mm to 70 mm, the breakthrough pressure that the alloy in the perforation could withstand rose from 6.989 kN to 9.779 kN, representing a 28.5% increase. When the plugging length further increased from 70 mm to 100 mm, the breakthrough pressure of the alloy in the perforation increased from 9.779 kN to 11.337 kN, with a 13.7% growth. When the rock perforation diameter was 8 mm, as the plugging length changed from 40 mm to 70 mm, the breakthrough pressure of the alloy in the perforation increased by 17.9%. When the plugging length extended from 70 mm to 100 mm, the breakthrough pressure of the alloy in the perforation increased by 6.9%. When the rock perforation diameter was 6 mm, during the increase in the plugging length from 40 mm to 70 mm, the breakthrough pressure of the alloy in the perforation increased by 8.1%. However, when the rock plugging length changed from 70 mm to 100 mm, the breakthrough pressure of the alloy in the perforation decreased by 3.3%. The results indicated that, as the ambient temperature increased, the variation trend of the breakthrough pressure became more gradual.

It could be seen from Figure 13 and Figure 14a,b that, with the gradual increase in ambient temperature, the change of compressive load of alloy plug was less significant.

**Figure 15:**
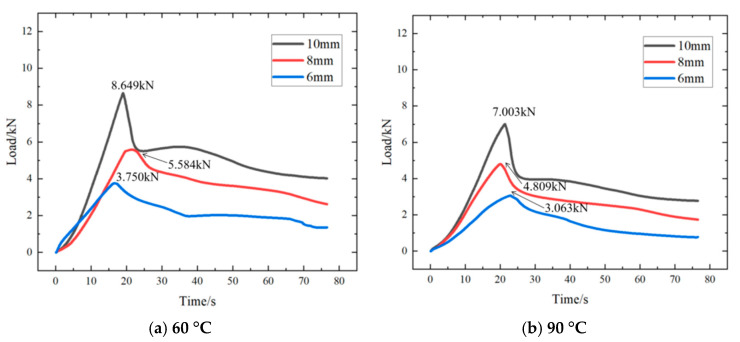
The curve of the load change with time.

In addition, the following figure is at the same rock perforation length (100 mm) and different ambient temperatures (30 °C (Figure 12), 60 °C (Figure 15a), and 90 °C (Figure 15b)), the alloy plug was pushed by external force (3 mm/min), and the curve of the load changing with time is shown in the figures.

As can be observed from Figure 12 and Figure 15a,b, with the rise in ambient temperature, the maximum load diminishes. However, at a constant temperature, the maximum load continues to increase as the diameter of the alloy plugging grows. Moreover, a positive correlation persists between the diameter of the alloy plugging and the maximum load.

In summary, under identical conditions (an ambient temperature of 30°C, a perforation diameter of 10 mm, and a length of 100 mm), the mechanical plugging performance of cement is inferior to that of the alloy. As the ambient temperature increases, the maximum load-bearing capacity of the alloy decreases. At a constant temperature, the maximum load-bearing strength of the alloy continues to increase with the growth of the alloy plugging diameter.

### 5.2. Effect of Ambient Temperature on Alloy Plugging Hydraulic Plug

The following Table 3 presents the experimental data of the hydraulic plug at different ambient temperatures when the length of the alloy plug in the rock was 100 mm.

**Figure 16:**
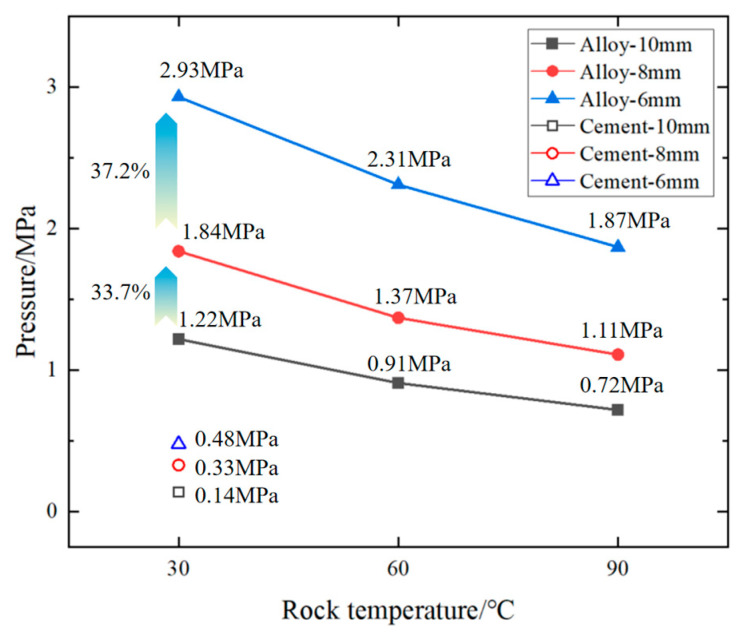
Effect of Sn58Bi alloy plugging and cement plugging on hydraulic plugging of rock.

Figure 16 shows the relationship among cement plugging (at 30 °C) and alloy plugging (at 30 °C, 60 °C, 90 °C), diameters (10 mm, 8 mm, 6 mm), and hydraulic plugging pressure (MPa) when the rock perforation length was 100 mm.

As can be deduced from Figure 16, under the same conditions, the hydraulic plugging performance of cement is less effective than that of the alloy. When the length of the alloy-plugged perforation is 100 mm, the higher the ambient temperature, the poorer the hydraulic plugging effect. Additionally, as the diameter of the perforation plugging increases, the hydraulic plugging performance deteriorates. When the alloy-plugging length is 100 mm and the ambient temperature is 30 °C, as the alloy-plugging diameter decreases from 10 mm to 8 mm, the hydraulic plugging pressure rises from 1.22 MPa to 1.84 MPa, representing a 33.7% increase. When the diameter further decreases from 8 mm to 6 mm, the hydraulic plugging pressure increases from 1.84 MPa to 2.93 MPa, representing a 37.2% growth.

## 6. Conclusions

In this paper, the properties and parameters of Sn58Bi alloy melt plugging in rock perforation have been studied through experiments, including the influence of ambient temperature, plugging diameter, and length of perforation on the properties of alloy plugging in rock; the obtained data provide theoretical support for the practical application of Sn58Bi alloy plugging rock perforation. The following conclusions are obtained:

(1) Under identical conditions and when the ambient temperature (30 °C, 60 °C, 90 °C) is lower than the melting point of the Sn58Bi alloy (138 °C), both the mechanical and hydraulic plugging performances of the alloy are superior to those of cement. The primary reason lies in the fact that the alloy undergoes a certain slight expansion upon solidification. This expansion contributes to a more effective plugging effect between the alloy and the rock perforation.
(2) As the ambient temperature rises, the internal structure of the Sn58Bi alloy undergoes alterations, and the bonding interface between the alloy and the rock perforation is also affected. This ultimately results in the degradation of the mechanical plugging performance of the alloy plug. Moreover, this performance degradation is associated with the diameter of the alloy plug. As the diameter of the alloy plug decreases, the micro-expansion effect induced by the alloy weakens. Consequently, the radial force between the alloy and the rock diminishes, which in turn leads to a further decline in the mechanical plugging performance of the alloy plug.
(3) At a constant alloy-plugging length, as the ambient temperature of the Sn58Bi alloy-plugged rock increases, the hydraulic plugging effect weakens. Conversely, as the diameter of the alloy-plugged pores decreases (from 10 mm to 8 mm and then to 6 mm), the hydraulic plugging performance of the alloy improves. Furthermore, with a reduction in the alloy-plugging diameter, the disparity in plugging performance becomes more pronounced.

## Figures and Tables

**Table 1:** Basic characteristics of Sn58Bi alloy [13,14].

Melting Point (°C)	Surface Tension (MPa·s)	Volume Change (Liquid to Solid)	Density /g × cm^−3^	Elastic Modulus /GPa	Tensile Strength /MPa	Elongation /25 °C
138	438	+0.77%	8.72	47.2	71.7	20.1%

**Table 2:** Alloy plugging in rock: mechanical push-out experimental data (Load/kN).

Rock Perforation Length	Ambient Temperature	Perforation Diameter 10 mm	Perforation Diameter 8 mm	Perforation Diameter 6 mm
100 mm	30 °C	11.377	7.924	5.706
	60 °C	8.649	5.584	3.750
	90 °C	7.003	4.809	3.063
70 mm	30 °C	9.779	7.377	5.517
	60 °C	7.546	5.486	3.612
	90 °C	6.830	4.668	2.897
40 mm	30 °C	6.989	6.059	5.068
	60 °C	6.605	5.330	3.325
	90 °C	6.530	4.578	2.687

**Table 3:** Experimental data of alloy plugging rock hydraulic plug (Pressure / MPa).

Ambient Temperature	Perforation Diameter 10 mm	Perforation Diameter 8 mm	Perforation Diameter 6 mm
30 °C	1.22	1.84	2.93
60 °C	0.91	1.37	2.31
90 °C	0.72	1.11	1.87

## Data Availability

The original contributions presented in the study are included in the article; further inquiries can be directed to the corresponding authors.

## References

[1] Wang H., Sun X. (2006). A Summary of Perforating Technology Development inside and outside of China. Explos. Mater..

[2] Chen C., Dai Y., Zhang H. (2014). Research on sealing technology of oil and water well. China’s New Technol. New Prod..

[3] Liu H., Wang F., Wang Y. (2014). Oil well perforation technology: Status and prospects. Pet. Explor. Dev..

[4] Reddy B.R., Xu Y., Ravi K., Gray D.W., Pattillo P. (2009). Cement-Shrinkage Measurement in Oilwell Cementing—A Comparative Study of Laboratory Methods and Procedures. SPE Drill. Complet..

[5] Liu Z.-X., Gao M., Zhang X.-M., Liang Y., Guo Y.-J., Liu W.-L., Bao J.-W. (2023). CCUS and CO_2_ injection field application in abroad and China: Status and progress. Geoenergy Sci. Eng..

[6] Saasen A., Wold S., Ribesen B.T., Tran T.N., Huse A., Rygg V., Grannes I., Svindland A. (2011). Permanent abandonment of a North sea well using unconsolidated well plugging material. SPE Drill. Complet..

[7] Jia Z., Shi B., Zhou H., Chen F., Meng X. (2017). Well Path Optimization Technique for Horizontal Wells in Reservoirs with Ultra—Low Permeability and Horizontal Fractures. Spec. Oil Gas Reserv..

[8] Carragher P., Fulks J. Well Abandonment Solutions Utilizing Bismuth and Thermite. Proceedings of the Offshore Technology Conference.

[9] Manataki A., Kontis P., Sangesland S. Investigation of the microstructure of bismuth alloy and its interaction with cement and steel casing. Proceedings of the ASME 2023 42nd International Conference on Ocean, Offshore and Arctic Engineering.

[10] Zhang H., Ramakrishnan T.S., Elias Q.K., Perez A. (2020). Evaluation of bismuth-tin alloy for well plug and abandonment. SPE Prod. Oper..

[11] Hmadeh L., Elahifar B., Sangesland S., Abrahamsen A.E. A Full Laboratory Study on the Physical and Mechanical Properties of a Bismuth Plug. Proceedings of the SPE/IADC International Drilling Conference and Exhibition.

[12] Hmadeh L., Jaculli M.A., Elahifar B., Sangesland S. (2024). Development of bismuth-based solutions for well plugging and abandonment: A review. Pet. Res..

[13] Zhang H., Ramakrishnan T.S., Elkady Y.M., Feng Y., Elias Q.K. (2020). Comparative Evaluation of Bismuth-Silver and Bismuth-Tin Alloys for Plug and Abandonment. SPE Drill. Complet..

[14] Liang B., Hu P., Ni Y., Feng G., Zhou J., Bai Y., Niu G., Niu W., Zhou Y. (2021). Experimental of mechanical properties and mechanism for theeutectic Sn-Bi alloys under different temperatures and strain rates loading. Funct. Mater..

[15] Carragher P., Fulks J. A New Look at Sealing with Bismuth and Thermite. Proceedings of the SPE Annual Technical Conference and Exhibition.

[16] Abdelal G.F., Robotham A., Carragher P. (2015). Numerical simulation of a patent technology for sealing of deep-sea oil wells using nonlinear finite element method. J. Pet. Sci. Eng..

